# Open-label placebos—a systematic review and meta-analysis of experimental studies with non-clinical samples

**DOI:** 10.1038/s41598-023-30362-z

**Published:** 2023-03-04

**Authors:** Lukas Spille, Johannes C. Fendel, Patrik D. Seuling, Anja S. Göritz, Stefan Schmidt

**Affiliations:** 1 Department of Psychology, Occupational Psychology, University of Freiburg, Freiburg, Germany; 2 Department of Psychosomatic Medicine and Psychotherapy, Medical Center - University of Freiburg, Faculty of Medicine, University of Freiburg, Freiburg, Germany

**Keywords:** Medical research, Outcomes research

## Abstract

The use of open-label placebos (OLPs) has shown to be effective in clinical trials. We conducted a systematic review and meta-analysis to examine whether OLPs are effective in experimental studies with non-clinical populations. We searched five databases on April 15, 2021. We conducted separate analyses for self-reported and objective outcomes and examined whether the level of suggestiveness of the instructions influenced the efficacy of OLPs. Of the 3573 identified records, 20 studies comprising 1201 participants were included, of which 17 studies were eligible for meta-analysis. The studies investigated the effect of OLPs on well-being, pain, stress, arousal, wound healing, sadness, itchiness, test anxiety, and physiological recovery. We found a significant effect of OLPs for self-reported outcomes (*k* = 13; standardized mean difference (*SMD*) = 0.43; 95% *CI* = 0.28, 0.58;* I*^*2*^ = 7.2%), but not for objective outcomes (*k* = 8; *SMD* =  − 0.02; 95% *CI* =  − 0.25, 0.21; *I*^*2*^ = 43.6%). The level of suggestiveness of the instructions influenced the efficacy of OLPs for objective outcomes (*p* = 0.02), but not for self-reported outcomes. The risk of bias was moderate for most studies, and the overall quality of the evidence was rated low to very low. In conclusion, OLPs appear to be effective when examined in experimental studies. However, further research is needed to better understand the mechanisms underlying OLPs.

## Introduction

The term ‘placebo’ is commonly defined as an inert substance or procedure^1,2^. Placebos come in many forms such as sugar pills, saline injections, sham surgery, or verbal interventions. The ‘placebo response*’* is best described as a biopsychosocial phenomenon that occurs subsequent to the application of a placebo^1,3^. Because changes in the placebo group may also be due to confounding factors such as regression to the mean or the natural tendency of the disease, comparison with a no treatment condition is necessary to detect and quantify the ‘placebo effect’^1,4^. In research, placebos are often applied as a control condition to determine a true intervention effect that is separate from influences that may be attributed to the psychosocial context^5–7^. The finding that people’s conditions often improve in placebo groups has given rise to a new research domain, focusing entirely on the placebo^1^. Aside from their scientific use, placebos are often administered in practical medicine to treat and/or appease patients^8^.

Placebos are mostly administered by physicians in a hidden way^1,9^. Concealment was long assumed to be an elementary component for the occurrence of the placebo effect^5^. However, this approach has been criticized for withholding the truth from participants; thus violating both the concept of informed consent and autonomy^10,11^. A potential solution to this problem is the use of *open-label placebos* (OLPs). OLPs are placebos that are administered openly without deception (i.e., subjects are told that they will receive a placebo). Research on OLPs dates back as early as 1965, when Park and Covi conducted a groundbreaking study on psychiatric outpatients^12^, who received placebos and were clearly informed that they were receiving only sugar pills with no active ingredient. The patients in this uncontrolled study improved significantly in both physician ratings and patient ratings. However, the topic had not been taken up for 45 years when in 2010, Kaptchuk et al. published a study on the use of OLPs in irritable bowel syndrome that attracted attention^13^. This sparked interest in OLPs and led to an increase in research activities. A recent meta-analysis by our research group has shown that OLPs are effective when used in clinical trials^14^. The authors included studies examining OLPs for a range of conditions including ADHD, depression, chronic lower back pain, irritable bowel syndrome, allergic rhinitis, cancer-related fatigue, and menopausal hot flushes, and found an overall medium-sized effect (SMD = 0.72) of OLPs compared to no treatment. All outcomes used for analysis were based on self-report.

In addition to clinical trials, it is also important to investigate the effects of OLPs in experimental studies in non-clinical, healthy populations. The efficacy of OLPs in non-clinical populations may differ substantially from that found in clinical trials, as there are specific factors that may contribute to the placebo response only in clinical trials. For example, patients suffering from chronic or refractory diseases may be more likely than healthy peers to place hope in novel interventions such as OLPs^15^. Investigating the extent to which OLPs work in healthy participants will help to understand the conditions and mechanisms underlying the effects of OLPs without the confounding influence of patient hope. Finally, this review may contribute to the consideration of OLPs as an alternative to the continued use of deceptive placebos in clinical as well as experimental research and to overcome the ethical concerns associated with them. However, a systematic summary of experimental studies of OLPs with healthy samples is yet to be published.

The aim of this study is to conduct a systematic review and meta-analysis to determine the efficacy of OLPs in experimental studies with non-clinical, healthy populations. Therein, OLPs are compared either to no treatment (NT) or to covert placebo (CP). Covert placebo refers here to a control condition in which the same physical treatment is given as in the OLP condition. However, the covert placebo is administered with an explanation that distracts participants’ attention from the dependent variable, for example, by giving a technical reason for the placebo’s use. This explanation thus acts as a ‘cover story’ for the intended effect on the dependent variable. We hypothesize that (1) OLPs are more effective as compared to NT or CP. Moreover, research has shown that OLPs are more effective the more suggestive the instructions that accompany the placebo’s administration^13,16,17^. Therefore, we hypothesize that (2) higher levels of suggestiveness are associated with greater OLP efficacy.

## Results

### Study selection

**Figure 1:**
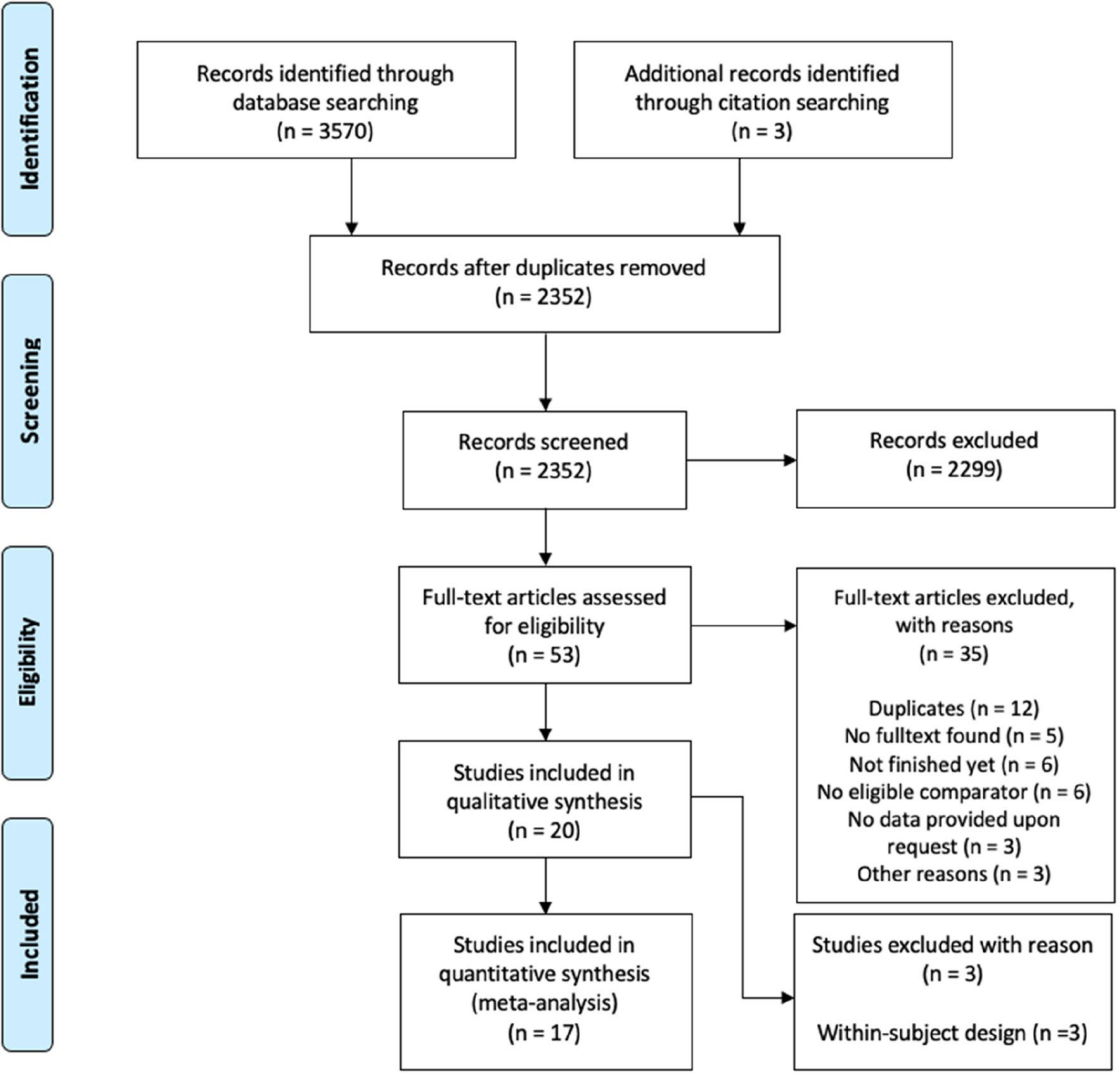
PRISMA flow diagram for studies selection. *Note.* In two cases, an individual article reported data on two independent experiments and was therefore considered as two studies. RCT—Randomized controlled trial.

Our search of five electronic databases on April 15, 2021 (see “Methods” section below) yielded 3573 records (Fig. 1). After removing duplicates, the remaining 2352 titles and abstracts were screened, and 53 full texts were assessed for eligibility. We included 18 eligible articles comprising 20 studies (1201 participants) in the systematic review. Two articles reported on two independent experiments, each experiment involving a different sample^18,19^. We included these experiments as individual studies in the analyses. An overview of the studies that we excluded after the full-text screening is in Table S1 (Supplementary Material). Three studies were excluded from the meta-analyses on account of their within-subject design^20–22^, leaving a total of 17 studies.

### Characteristics of studies, participants, and interventions

All of included studies were Randomized Controlled Trials (RCTs), of which three were crossover studies^20–22^ and 17 were parallel-group studies^16,18,19,23–34^. In two parallel-group studies, a balanced placebo design was used^33,34^. Three studies used a CP control condition^18,28^, and all other studies used a NT control condition. Five studies included more than one OLP condition^16,19,23,25^. All studies were reported in English and published between 2001 and 2021. Nine studies were conducted in Germany^19,24,25,29–32,34^, four in the United States^18,20,28^, two in Switzerland^16,22^, two in Australia^23,33^, and one each in New Zealand^26^, the Netherlands^27^, and Brazil^21^.

Overall, 585 participants received an OLP intervention while 535 participants served as controls. In addition, 81 participants took part in crossover studies and therefore underwent both conditions. The sample sizes of individual studies ranged from 21 to 199.

Five studies investigated the effect of an OLP on pain^16,22,25,28,29^. Three studies examined the influence of OLPs on well-being^19,23^, three on stress^18,31^, and two on arousal^32,34^. OLPs have also been studied in relation to wound healing^26^, sadness^24^, itchiness^27^, test anxiety^30^, cycling performance^21^, physiological recovery^33^, and muscle strength and fatigue^20^. A wide range of placebos was used in the studies, including a nasal spray^18,24,29^, pills^23,26,30,31^, creams^16,25,28^, capsules^20,21^, bottled water^19^, decaffeinated coffee^32,34^, verbal suggestions^27^, acupuncture^33^, and intravenous injection^22^. The degree of instructional suggestiveness was similarly distributed across studies, with five studies receiving a rating of 0 regarding the degree of instructional suggestiveness^24,29,32–34^, six studies receiving a rating of 1^18–20,27^, four studies receiving a rating of 2^21,25,26,28^, and five studies receiving a rating of 3^16,22,23,30,31^. Study characteristics are in Table 1. Detailed instructions accompanying the administration of the OLPs are in Table S2.

**Table 1:** Characteristics of included studies.

Authors	Year	Country	Context	N	Intervention	Control	Suggestiveness rating of instruction	Self-reported outcome used for meta-analysis	Objective outcome used for meta-analysis
El Brihi et al.^23^	2019	AU	Well-being	92	OLP (*n* = 61)	NT (*n* = 27)	3	Depression anxiety stress scale 21 (DASS-21) Subjective health complaints inventory (SHC) Pittsburgh sleep quality index (PSQI) Warwick-Edinburgh Mental Well-being Scale (WEMWBS)	Not applicable
Glombiewski et al.^24^	2019	GER	Sadness	128	OLP (*n* = 32)	NT (*n* = 32)	0	Sadness subscale from the Positive and Negative Affect Schedule-Expanded Form (PANAS-X)	Not applicable
Guevarra et al. a)^18^	2020	US	Emotional distress	62	OLP (*n* = 29)	CP (*n* = 33)	1	Emotional distress on a nine-point Likert scale	Not applicable
Guevarra et al. b)^18^	2020	US	Emotional distress	198	OLP (*n* = 99)	CP (*n* = 99)	1	Not applicable	Sustained late positive potential
Kube et al.^25^	2020	GER	Pain	100	OLP-E (*n* = 25)	NT (*n* = 25)	2	Pain intensity (VAS) Pain unpleasantness (VAS)	Pain tolerance (°C)
Locher et al.^16^	2017	CH	Pain	151	OPR+ (*n* = 37)	NT (*n* = 40)	3	Pain intensity (VAS) Pain unpleasantness (VAS)	Pain tolerance (°C)
Mathur et al.^26^	2018	NZ	Wound healing	65	OLP (*n* = 32)	NT (*n* = 33)	2	Not applicable	Percentage area of the wound healed after 7 and 10 days
Meeuwis et al.^27^	2017	NL	Itch	92	OLP (*n* = 45)	NT (*n* = 46)	1	Itch (NRS)	Not applicable
Mundt et al.^28^	2016	US	Pain	75	OLP (*n* = 25)	CP (*n* = 25)	2	Pain intensity (VAS)	Not applicable
Rathschlag and Klatt a)^19^	2021	GER	Well-being	68	OPR+ (*n* = 18)	NT (*n* = 16)	1	Acute Recovery and Stress Scale (ARSS, all of the 8 subscales) Questionnaire for Assessing Subjective Physical Well-Being (FEW-16, all of the 4 subscales)	Not applicable
Rathschlag and Klatt b)^19^	2021	GER	Well-being	75	OPR+ (*n* = 18)	NT (*n* = 19)	1	Acute Recovery and Stress Scale (ARSS, all of the 8 subscales) Questionnaire for Assessing Subjective Physical Well-Being (FEW-16, all of the 4 subscales	Not applicable
Rief and Glombiewski^9^	2012	GER	Pain	134	OLP (*n* = 41)	NT (*n* = 20)	0	Not applicable	Pain threshold (°C)
Saunders et al.^21^	2019	BRA	Cycling time trial	28	OLP (*n* = 28)	NT (*n* = 28)	2	Not applicable	Not included in analysis
Schaefer et al.^30^	2019	GER	Test anxiety	58	OLP (*n* = 31)	NT (*n* = 27)	3	Brief German test anxiety inventory (PAF) Questionnaire for Measuring Resources and Self-Management Skills (FERUS)	Not applicable
Schaefer et al.^31^	2021	GER	Stress	53	OLP (*n* = 24)	NT (*n* = 29)	3	State dimension of the State-Trait-Anxiety-Inventory (STAI-S) Positive and Negative Affect Scale (PANAS) Current stress (VAS)	Not applicable
Schneider et al.^32^	2006	GER	Arousal	45	OLP (*n* = 15)	NT (*n* = 15)	0	Multi-dimensional Well-Being Questionnaire (all of the 3 dimensions)	Systolic blood pressure (mmHg) Diastolic blood pressure (mmHg) Heart rate (bpm) Reaction time (ms)
Schneider et al.^22^	2020	CH	Pain	32	OLP (*n* = 32)	NT (*n* = 32)	3	Not included in analysis	Not applicable
Swaffordet al.^20^	2019	US	Muscular strength and fatigue	21	OLP (*n* = 21)	NT (*n* = 21)	1	Not included in analysis	Not included in analysis
Urroz et al.^33^	2016	AU	Physio-logical recovery	60	OLP (*n* = 12)	NT (*n* = 12)	0	Not applicable	Systolic blood pressure (mmHg) Diastolic blood pressure (mmHg) Heart rate (bpm) Volume of oxygen consumption in ml·min Respiratory rate (breaths/min) Blood lactate (mmol/l)
Walach et al.^34^	2001	GER	Arousal	156	OLP (*n* = 41)	NT (*n* = 37)	0	Basle Well-Being Scale	Systolic blood pressure (mmHg) Diastolic blood pressure (mmHg) Heart rate (bpm)

*OLP* open-label placebo, *NT* no treatment, *CP* covert placebo, *AU* Australia, *BRA* Brazil, *CH* Switzerland, *GER* Germany, *NZ* New Zealand, *US* United States, *VAS* visual analogue scale, *NRS* numeric rating scale, *N* total sample size.

For reasons of clarity and comprehensibility, only the intervention and control groups relevant to this review were listed with their corresponding group sizes. Four studies implemented multiple OLP groups, which is why the specific name of the group used for this study was provided here.

### Risk of bias within studies

There was little variation among studies in risk of bias (see Figure S1). Seventeen studies (85%) were rated as having “some concerns”^16,18–25,27–33^, two studies (10%) as “low risk of bias”,^26,34^ and one study (5%) as “high risk of bias”^18^. The randomization process was not adequately described in some studies^18–22,29^. Others presented biases arising from the selection of the reported results^16,18–22,24,25,27–33^ or unblinded outcome assessors^18,19,22–25,28,30,31^.

### Data synthesis and analyses

**Figure 2:**
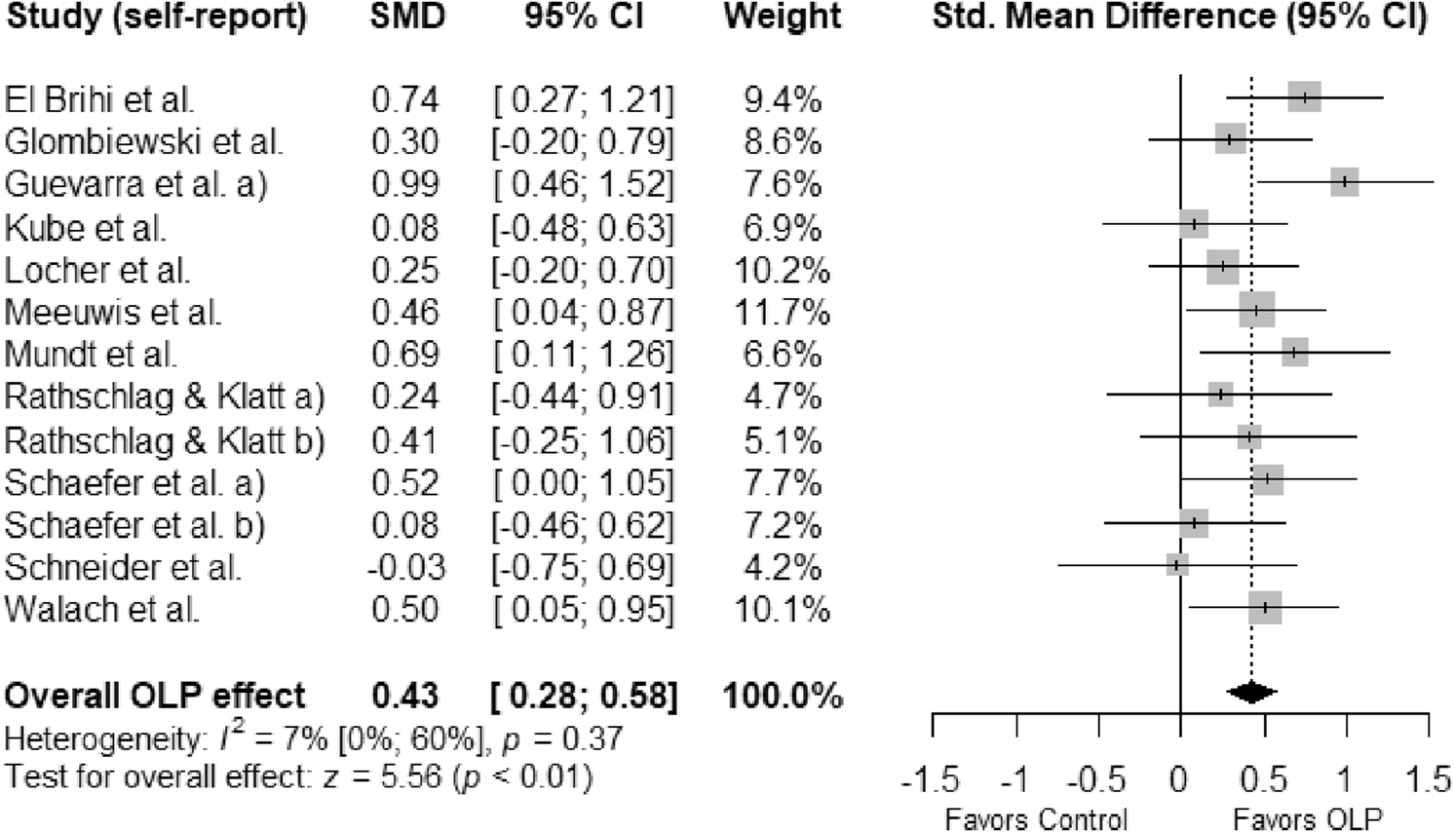
Forest plot of the effects of open-label placebos vs. no treatment or covert placebo on self-reported outcomes in experimental studies with non-clinical, healthy individuals. *Note.* Studies were weighted using the inverse-variance method. The size of grey squares represents study weight. Whiskers represent the 95% *CI*. The overall *SMD* is shown as a black diamond. *CI*—confidence interval, *SMD*—standardized mean difference.

Thirteen studies (772 participants, 401 in the experimental group and 371 controls) were included in the meta-analysis on self-reported outcomes: Analysis revealed a significant positive effect of OLPs compared to NT or CP (*SMD* = 0.43; 95% *Cl* = 0.28, 0.58; *p* < 0.01; Fig. 2). Heterogeneity was low and non-significant: χ^2^ (12) = 12.94, *p* = 0.37, *I*^*2*^ = 7.2%.

**Figure 3:**
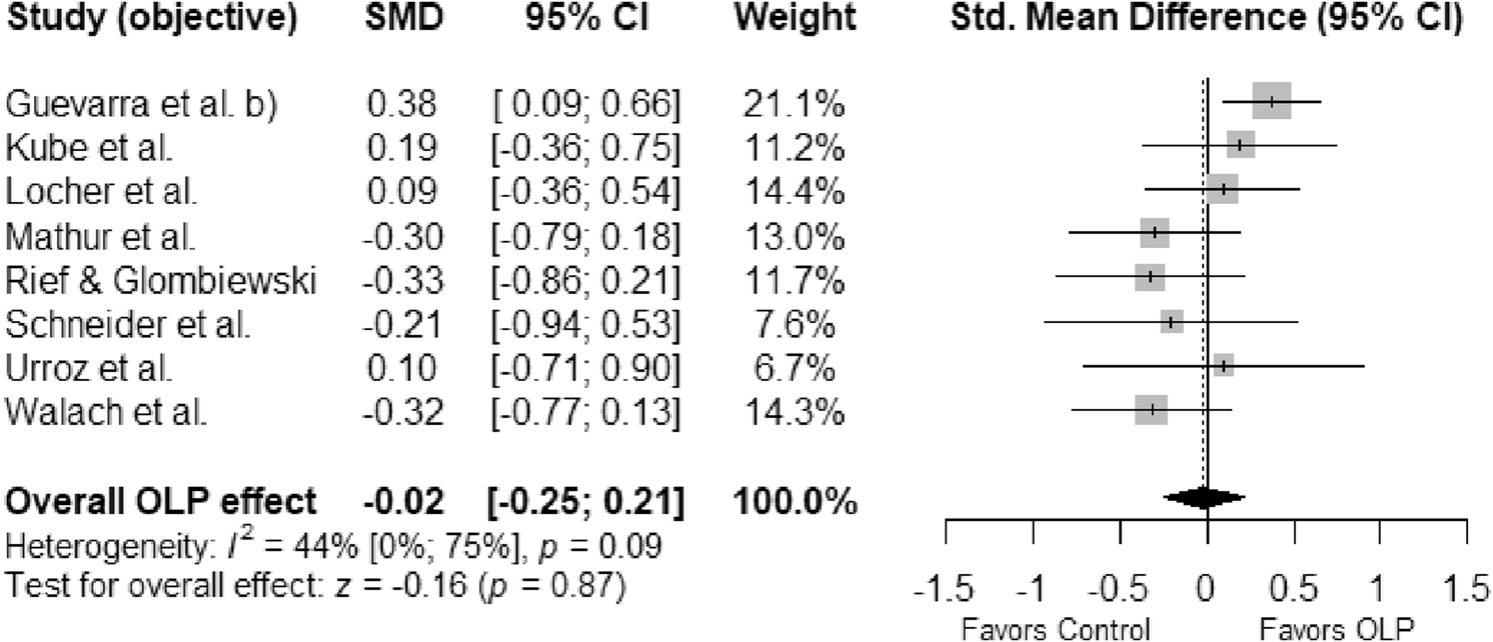
Forest plot of the effects of open-lapel placebos vs. no treatment or covert placebo on objective outcomes in experimental studies with non-clinical, healthy individuals. Note. Studies were weighted using the inverse-variance method. The size of grey squares represents study weight. Whiskers represent the 95% *CI*. The overall *SMD* is shown as a black diamond. *CI*—confidence interval, *SMD*—standardized mean difference.

Eight studies (583 participants, 302 in the experimental group and 281 controls) were included in the meta-analysis on objective outcomes: We did not find a significant effect of OLP compared to NT or CP (*SMD* =  − 0.02; 95% *Cl* =  − 0.25, 0.21; *p* = 0.87; Fig. 3). Heterogeneity was low and non-significant: χ^2^ (7) = 12.40, *p* = 0.09, *I*^*2*^ = 43.6%. However, the results of the heterogeneity tests should be considered with caution due to low power^35^.

There were five studies with more than one OLP group^16,19,23,25^, necessitating the selection of data for the analyses. With one study^23^ we chose to combine the data from both OLP groups, as they differed only in the number of placebos given to the participants. With the remaining four studies that had more than one OLP group^16,19,25^, we followed our approach of selecting whichever group had the most suggestive instructions (i.e., those that included the most statements from Kaptchuk et al.^13^). Specifically, with one study that had more than one OLP group^16^, we used the data from the group receiving an open-label placebo with rationale (OPR+) instead of the group receiving open-label placebo without rationale (OPR −). With another study that had more than one OLP group^25^, we used the data from the group in which expectancies were evoked (OLP-E) rather than the group in which hope was induced (OLP-H) as instructions in the OLP-E group were more closely aligned with Kaptchuk et al.^13^. Two further studies had three OLP conditions^19^. From these studies we used the data from the OPR + group, as the other groups either did not receive a placebo rationale (OPR group) or received an additional treatment in the form of a relaxation and imagination exercise (OPR++ group), which would have limited comparability with the placebo groups in the other included studies.

We obtained change scores for all studies except for one^18^, for which only post-intervention scores were reported due to lack of baseline measurements. Another study reported post-intervention values^16^, but we considered these as change scores, as they were adjusted for the corresponding baseline scores.

### Subgroup analyses

For self-reported outcomes, the subgroups with different levels of suggestiveness did not differ significantly (*Q*(3) = 1.02; *p* = 0.80; Table S3). For objective outcomes, the subgroups differed significantly (*Q*(3) = 9.49; *p* = 0.02). Specifically, the subgroup representing studies in which only one suggestive statement was communicated showed a significant OLP effect (*p* = 0.009), while the other subgroups did not (all *p*s > 0.05). However, only eight studies were included in this analysis, with two subgroups involving only one study each. Therefore, these results should be interpreted with caution.

Exploratory subgroup analyses examining the influence of the type of control condition (CP or NT) on OLP efficacy revealed that subgroups differed significantly for both self-reported (*Q*(1) = 5.26; *p* = 0.02; see Table S4) and objective outcomes (*Q*(1) = 8.14; *p* < 0.01). In both analyses, the subgroup representing studies with CP controls descriptively yielded a larger effect size than the subgroup of studies with NT controls (see Table S4). However, the number of studies within subgroups was imbalanced. In the analysis of self-reported outcomes, 11 studies were in the subgroup of NT controls and only two studies were in the subgroup of CP controls. As for the analysis of objective outcomes, seven studies were in the subgroup of NT controls and only one was in the subgroup of CP controls. Because of the paucity of studies with CP controls, these results can only provide preliminary evidence and should be interpreted with great caution. Another exploratory analysis examining whether the OLP effect differed between studies involving completely healthy individuals and studies involving individuals with subclinical complaints (e.g., test anxiety) revealed no significant difference (*Q*(1) = 0.68; *p* < 0.41; see Table S5). Again, due to the limited number of studies, these results should be interpreted with caution.

### Reporting bias

**Figure 4:**
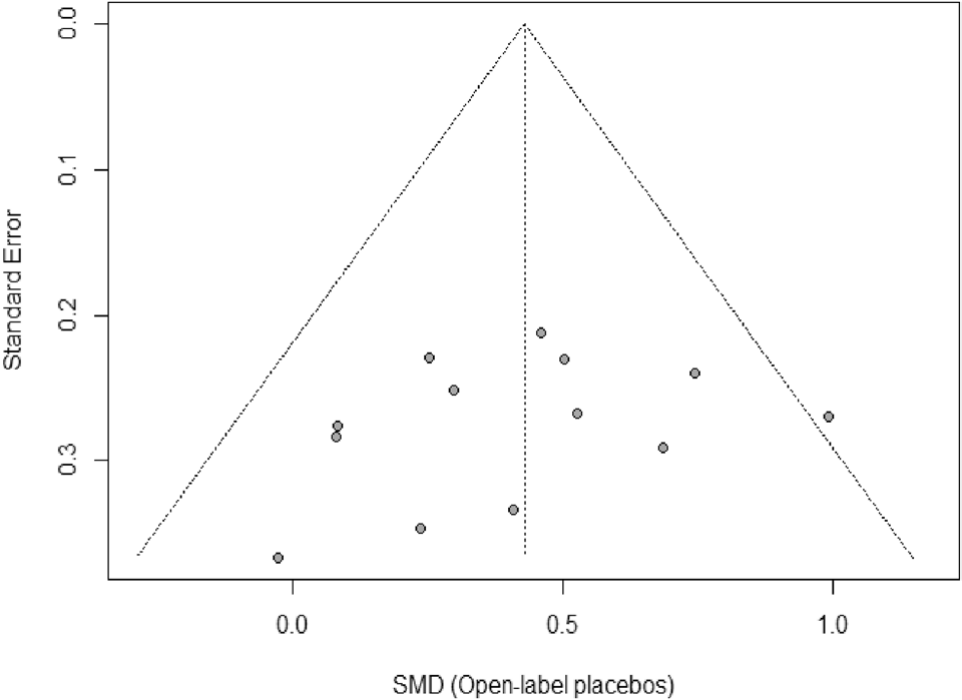
Funnel plot of self-reported outcomes. *Note.* Effect estimates (*SMD*) from individual studies are plotted against their standard error (*SE*). The dotted lines of the triangle represent the area in which 95% of studies are expected to be located if both publication bias and heterogeneity are absent.

**Figure 5:**
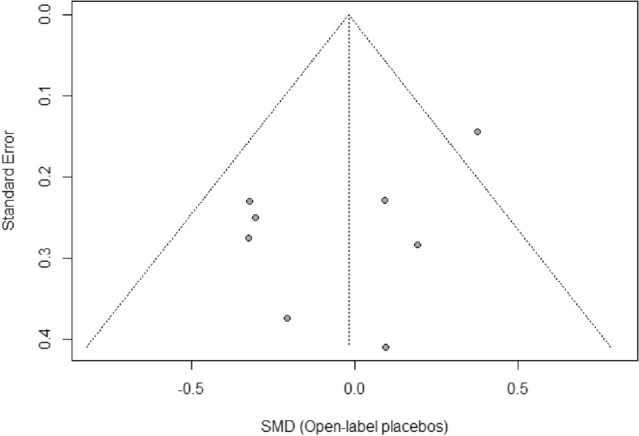
Funnel plot of objectively recorded outcomes. Note. Effect estimates (*SMD*) from individual studies are plotted against their standard error (*SE*). The dotted lines of the triangle represent the area in which 95% of studies are expected to be located if both publication bias and heterogeneity are absent.

The visual inspection of the funnel plot as well as Egger’s regression test indicated no evidence of publication bias either for self-reported outcomes (*intercept* =  − 1.85; 95% *Cl* − 5.51, − 1.81; *p* = 0.34; Fig. 4), or for objective outcomes (*intercept* =  − 2.38; 95% *Cl* − 4.97, − 0.22; *p* = 0.12; Fig. 5). For objective outcomes, however, results should be interpreted with caution as Egger's regression test had low power^36^.

### Certainty of evidence

The overall quality of the evidence was assessed with the Grading of Recommendations Assessment, Development and Evaluation (GRADE)^37^ approach. Using this tool, the overall quality of evidence was rated as low to very low. Specifically, the overall quality of evidence was rated as low for objective pain, self-reported distress, and self-reported well-being. The overall quality of evidence was rated as very low for self-reported pain and the sub-clusters of physiological outcomes. Details on the GRADE ratings are in Table S6.

## Discussion

Although OLPs were studied as early as 1965, broader interest in this topic has emerged only recently, after several clinical trials of this particular type of placebo application were published^12–14^. This provided the impetus for more research on OLPs, both in clinical and in experimental studies. We conducted a systematic review and meta-analysis to investigate whether the OLP effect found in clinical trials holds true in experimental studies with non-clinical, healthy individuals. We included 20 studies, of which 17 were suitable for meta-analyses. Thirteen studies analyzed the OLP effect with self-reported outcomes and eight studies with objective outcomes. The results of the meta-analyses revealed a small to medium OLP effect for self-reported outcomes and no OLP effect for objective outcomes. Subgroup analyses revealed that the level of suggestiveness of the instructions influenced the efficacy of OLPs for objective outcomes, but not for self-reported outcomes. However, due to the small number of studies, these results regarding suggestiveness should be viewed with caution. An exploratory subgroup analysis suggested that the use of CP as a comparator resulted in greater OLP efficacy as compared to NT. However, because of the limited number of studies using CP controls, these results are tentative and should be interpreted with caution.

Our finding of a significant OLP effect for self-reported outcomes in healthy individuals is consistent with a meta-analysis on clinical trials^14^. The aggregated *SMD* of that meta-analysis on clinical trials was larger than what we found in experimental studies with non-clinical, healthy individuals (*SMD* 0.72 vs. 0.43). There is reason to believe that patients suffering from clinical symptoms or medical conditions show higher placebo susceptibility because of plausible differences in the therapeutic encounter and contextual aspects surrounding the placebo administration in clinical trials compared to experimental ones. In the clinical setting, for example, emphasis is placed on attentive and supportive patient care. This may create a therapeutic bias leading patients to expect improvement^3^. As mentioned earlier, people seeking treatment often hope for relief^15^. Thus, there may be fundamental differences between patients and healthy individuals in the mechanisms that contribute to the placebo effect such as motivation and expectation^1^. Consequently, there might also be differences in OLP effect between experimental studies on completely healthy participants versus participants with subclinical complaints. Such subclinical samples are also present in our study, for example, Schaefer et al.^30^ who investigated the influence of OLPs on test anxiety. However, in the explorative subgroup analysis comparing experimental studies involving completely healthy participants with studies involving individuals with subclinical complaints we could not find any difference in the OLP effect.

Regarding the differences between self-reported and objective outcomes, our finding of a null effect for objective outcomes raises the question of whether OLPs and deceptive placebos have the same pattern of effect, as changes in objective outcomes have been repeatedly demonstrated in studies using deceptive placebos^38^. One might therefore hypothesize that OLPs, unlike deceptive placebos, do not entail biological changes. However, Kaptchuk & Miller^3^ emphasize that also deceptive placebos primarily affect self-reported and self-appraised symptoms. Further studies comparing the effects of OLPs with deceptive placebos on objective outcomes are needed to clarify this issue.

The level of suggestiveness of the instructions showed an association with the efficacy of OLPs only for objective outcomes. This association was contrary to our hypothesis. In this analysis the one subgroup that showed a significant OLP effect consisted of only one study (Table S3), which had a sample size three times as large as the average study assessing objective outcomes and therefore had a substantially higher weight in the analysis relative to the others. Thus, this finding should be interpreted very cautiously. Overall, the observed results do not confirm our hypothesis that the OLP effect increases with higher instructional suggestiveness. However, this may change with the future publication of larger OLP studies that would make a reassessment of this issue possible.

This review has several strengths. First, two researchers performed the screening processes and the application of the assessment tools, ensuring high quality and reliability. Second, we used an established search strategy^14^, increasing the comparability with other reviews on OLPs. Third, we did not use any date or language restrictions and searched as many as five electronic databases to be comprehensive. Moreover, in both meta-analyses, the heterogeneity was low, and the statistical examination of funnel plot asymmetry revealed no indication of publication bias.

This review also has a number of limitations. First, the number of studies is small, and therefore, the stability of the results might be low. Second, the variability of outcomes in the included studies was large and unbalanced. While most studies were conducted in the context of pain research or well-being, only a few others were conducted in other contexts. This limits the generalizability of the findings and impedes the derivation of specific practical implications for the application of OLPs. The fact that the overall quality of evidence, as assessed by the GRADE approach, was low to very low underscores this point. Third, the variability of investigators of OLPs in experimental studies is limited, and some authors have been involved in multiple publications, highlighting the need for further independent studies and replications. Fourth, to assess instructional suggestiveness we used an ad hoc developed tool based on the four suggestiveness statements by Kaptchuk et al.^13^. We followed this procedure because the instructions of most OLP studies are based on these four statements and because studies in which the instructions deviated slightly from Kaptchuk's framework were easily able to be subjected to this taxonomy. However, in isolated cases such as Meeuwis et al.^27^, there were instructions that deviated to a greater extent from the statements by Kaptchuk et al.^13^, indicating that an assessment based on our approach might not have captured all aspects of suggestiveness. Fifth, we used the RoB 2 to assess the methodological quality of the included studies. The application of the RoB 2 in the context of OLPs required an adaptation of the tool. Owing to the specifics of non-deceptive placebos, knowledge of the intervention received cannot be separated from the open-label placebo effect^14^. Therefore, we chose not to rate the knowledge of the intervention received as risk of bias. Future research would benefit from revised instruments that are adapted to the character of OLP interventions. Finally, one study was rated as having a high risk of bias. This study showed the largest effect size of all studies included in the meta-analysis of self-reported outcomes. Although the chi-squared test for heterogeneity was not significant in the analysis of self-reported outcomes and the *I*^2^ statistic indicated negligible heterogeneity, this high-risk study may have inflated the assessment of the overall effect due to methodological flaws.

This review adds to the evidence that OLPs offer the possibility of improving subjective symptoms without the need to lie about the placebo or to take active agents. Having said this, the opinions of many physicians towards OLPs differ greatly^39^. Patients, on the other hand, seem more open to this novel use of placebos. For example, in a study of placebo acceptability in patients with chronic pain, respondents indicated that they preferred open-label placebos to deceptive placebos^40^. However, there are also studies suggesting the opposite, that is, patients preferring deceptive over open-label placebos^41^. The patients' desire for transparency and right to informed consent aligns with the calls of leading placebo researchers who oppose the use of deceptive placebos in clinical practice^42^. Based on their confirmed efficacy in initial studies and low side effects, we suggest identifying circumstances in which OLPs might be preferable to deceptive placebos even in clinical settings, given that deceptive placebos might be ethically questionable in some circumstances.

The results of this systematic review and meta-analysis to examine the effect of OLPs in experimental studies with non-clinical, healthy individuals suggest that OLPs are effective for self-reported outcomes, but not for objective outcomes. The degree of instructional suggestiveness seems to influence the efficacy of OLPs only for objective outcomes, but not in the direction it was expected. These findings need to be confirmed in future research based on a larger number of primary studies. This would also allow for an adequate statistical investigation of the influence of different control conditions on the efficacy of OLPs.

## Methods

We preregistered this review at the Open Science Framework (OSF) on April 12, 2021 (https://doi.org/10.17605/OSF.IO/4CAFQ). The review adhered to the checklist of the Preferred Reporting Items for Systematic Reviews and Meta-Analyses (PRISMA)^43^.

### Eligibility criteria

Eligible studies had to meet the following criteria: (1) *Population*: We included studies with non-clinical populations. Studies with clinical populations were excluded. (2) *Intervention*: We included OLP interventions regardless of their specific application. (3) *Comparison*: We considered either a no-treatment control condition (NT) or covert placebo (CP) control condition. (4) *Outcome*: We included studies that measured the efficacy of OLPs on any given scale. Since the aim of this study was to investigate the effect of OLPs on a meta-level (i.e., across various outcomes), we did not apply restrictions to the types of outcomes. (5) *Design*: We included RCTs and excluded all other study designs.

### Information sources and search strategy

We screened five electronic bibliographic databases comprising all entries from database inception to April 15, 2021. We did not apply any language restrictions. We searched for studies using Medline via PubMed (1965 to April 15, 2021), PsycINFO via EBSCO (1967 to April 15, 2021), PSYNDEX via EBSCO (1977 to April 15, 2021), Web of Science Core Collection (1945 to April 15, 2021), and The Cochrane Central Register of Controlled Trials (CENTRAL, The Cochrane Library, Wiley), Issue 4 of April 12, 2021. Due to its composite nature, CENTRAL does not have an inception date. However, we did not apply any date restrictions and used the latest issue available. In addition, we screened the Journal of interdisciplinary placebo studies DATABASE (JIPS, https://jips.online/).

We used a search strategy similar to von Wernsdorff et al.^14^. The search terms served the purpose of describing the OLP intervention in more detail. Therefore, in addition to terms such as "placebo", we used synonyms for "open-label", such as “non blind” or “without deception”. Since the aim of this study was to investigate the effect of OLPs on a general level, we did not specify outcomes and control conditions in the search strings. In addition, we used wildcards and variant forms of spelling to find as many studies as possible. The search strings are in Tables S7, S8, S9, S10, S11. Slight variations between the search strings are due to different proximity operators across the databases. We compiled all records identified in the databases in the reference management software Zotero 5.0.96.2 (Corporation for Digital Scholarship, Vienna, Virginia) and removed duplicates. We conducted both backward and forward citation searches of all included studies and important reviews on OLPs^14,44^ using Web of Science and PsychINFO.

### Study selection and data extraction

Two researchers (LS and PDS) independently screened titles, abstracts, and full texts for inclusion. Title and abstract screening were carried out using the systematic review software Rayyan (Rayyan QCRI, Doha, Qatar). Disagreements were resolved through discussion. If no consensus could be reached, JCF and SS were consulted. The chance-corrected agreement between raters after the full text screening was substantial (*κ* = 0.62). In cases where eligible studies did not report the necessary information to compute effect sizes, we contacted the authors of the studies. If the authors did not respond or were unable to provide the data, these studies were excluded.

The same two researchers, who selected the studies, independently extracted the data. Again, disagreements were resolved through discussion. We extracted data on: author, year, country of trial, study design, sample size, control condition, intervention characteristics, the exact wording of instructions given to the participants, as well as the type and number of outcomes into a spreadsheet. For outcomes, we extracted the means, sample sizes, and standard deviations. If reported, this was done for change scores, otherwise for both pre- and post-intervention scores. For studies where only the standard error was reported, we transformed the standard error into the standard deviation according to the procedure outlined in the Cochrane Handbook^45^.

As stated in the preregistration, we extracted the primary outcome as specified in the individual studies. If multiple primary outcomes were specified in the individual studies, we extracted all primary outcomes. If no outcome was designated as primary, we extracted all outcomes. For studies that included multiple control conditions we only extracted data on the OLP condition and the corresponding comparator (i.e., NT or CP).

### Study risk of bias assessment

We used the revised Cochrane risk of bias tool for randomized trials (RoB 2) to assess the risk of bias in primary studies. Five domains of bias are assessed using the RoB 2, namely biases arising from (1) the randomization process, (2) deviations from intended interventions, (3) missing outcome data, (4) measurement of the outcome, and (5) selection of the reported result^46^. Ratings for each domain range from “low risk of bias”, to “some concerns”, to “high risk of bias”. Finally, the ratings of the individual domains are aggregated into an overall rating, which in most cases is equivalent to the worst rating in any of the domains^46^.

Given the specific context of OLP, we agree with von Wernsdorff et al.^14^ that a lack of blinding of participants should not result in an increased risk of bias rating. They argue that knowledge of one's group assignment is imperative and cannot be separated from the placebo effect in this particular intervention. Thus, we decided to rate the risk of bias in the domains (2) and (4) (i.e., the risk of bias due to unblinding) as not worse than “some concerns”. Risk of bias assessments were carried out independently by LS and PDS, with discrepancies resolved through discussion with JCF and SS.

### Data synthesis and analyses

Since knowledge of the received intervention might influence self-reported outcomes, we conducted two separate meta-analyses, one for self-reported outcomes and one for objectively recorded outcomes (i.e., physiological or behavioral variables). The meta-analyses were conducted using the meta package of R, version R 4.0.3. Since all studies reported continuous data, we chose the standardized mean difference (*SMD*) as the summary outcome. We used *Hedges’ g*, which corrects for small sample bias^47^. When both pre- and post-intervention values were reported, we first calculated change scores by subtracting pre-intervention from post-intervention scores. We then standardized the difference in change scores between groups using the pooled pre-intervention *SD* to calculate the corresponding *SMD*s.

If there were multiple outcomes within one study, we calculated SMDs for all of these outcomes and averaged them^48,49^. This approach ensured that there was no bias due to selective choice of outcome depending on effect size and conformity to the hypothesis.

When there were multiple OLP conditions within a study, we proceeded as follows: Our primary goal was to obtain the maximum OLP effect that could be realized experimentally. Since we assumed that suggestive instructions would amplify the placebo effect, we always chose whichever condition was most suggestive. This was operationalized by selecting the condition where most of the instructional statements from Kaptchuk et al.^13^ were utilized. Kaptchuk et al.^13^ were among the first to conduct a clinical trial of OLP and used a rationale (i.e., statements explaining the placebo effect) of four statements with positive framing to optimize placebo response. These statements imply 1) that the placebo effect is powerful, 2) that the body is automatically responding to placebos, 3) that it does not require a positive attitude, and 4) that taking the placebo faithfully is crucial. This or similar rationales were applied by many other researchers^14^.

Studies with crossover designs were not included in the meta-analyses as the coefficients required for the computation of the effect sizes were not reported. An alternative approach of analyzing crossover studies is to handle study groups as if they were parallel groups. However, this approach is not recommended by Cochrane as this may lead to a unit-of-analysis error^50^.

Once the effects of the individual studies were calculated, they were aggregated into an overall *SMD*. We employed a random effects model by applying the inverse-variance weighting method^47^. To correct for differences in the direction of the scale, the means of some studies were multiplied by − 1^50^. Heterogeneity between studies was assessed using the chi-square test and the *I*^2^ statistic. *I*^2^ values above 25% are interpreted as low, above 50% as moderate, and above 75% as high heterogeneity^51^.

We conducted subgroup analyses to examine the influence of the number of instructions given alongside the OLPs on the efficacy of OLPs. We hypothesize that the statements in the instruction create expectations that, in turn, may elicit placebo effects. We refer to this process as suggestiveness. To assess the degree of the suggestiveness of the instructions in OLPs, we developed a tool based on the four statements applied by Kaptchuk et al.^13^. These statements are given along with the administration of the open-label placebos. However, the placebos in most experimental studies included in our review were administered only once and under the supervision of an experimenter. Therefore, we omitted the fourth statement and formed four subgroups depending on the number of statements utilized in the instructions (ranging from 0 = “no statement utilized” to 3 = “all statements utilized”), with higher values indicating greater suggestiveness. We believe this approach to be reasonable, as many studies investigating OLPs have adopted the instructions from Kaptchuk et al.^13^ and varied the number of statements implemented in the instructions. For the subgroup analyses, we first calculated the pooled effect for each subgroup and then used a *Q* test to examine whether effect sizes differed between subgroups^52^.

We also conducted exploratory subgroup analyses to examine (1) whether the efficacy of OLPs differed depending on the control condition used (i.e., NT or CP) and (2) whether the efficacy of OLPs differed in lab studies with healthy participants compared to clinically oriented studies involving individuals with subclinical complaints (e.g., test anxiety). We used the same statistical procedures as before. However, these exploratory analyses were specified a posteriori and therefore not reported in the preregistration.

All tests were two-tailed.

### Reporting bias assessment

We assessed publication bias by visually inspecting funnel plots for asymmetry. In funnel plots, the *SMD*s of the individual studies are plotted against their standard error. In addition, we carried out a statistical assessment of funnel plot asymmetry using Egger's regression test, which regresses the SMDs against their standard error^53^. We did not assess the risk for time-lag bias, as research on OLPs is in its early stages and the interest in non-clinical, healthy populations has arisen only recently.

### Certainty assessment

We used the Grading of Recommendations Assessment, Development and Evaluation (GRADE)^37^ approach to assess the overall quality of the evidence. At the beginning of the assessment process, the overall quality of an RCT is rated as high and can subsequently be down- or upgraded based on eight dimensions: (1) risk of bias, (2) inconsistency, (3) imprecision, (4) indirectness, (5) publication bias, (6) dose response, (7) large effects, and (8) confounding. Based on the ratings of each dimension, the overall quality of evidence is rated as “high”, “moderate”, “low”, or “very low”. GRADE is performed for specific outcomes. However, due to the large number of different outcomes, we decided to form five clusters, in which similar outcomes were grouped together: self-reported pain, objective pain, self-reported positive well-being, self-reported distress, and physiological outcomes. For physiological outcomes, we formed three sub-clusters, each containing a single study, to account for the heterogeneity in physiological outcomes. In our approach, a study may be represented in several clusters due to different outcome variables, but in each cluster only once. Assessments were conducted by two independent raters (LS and PDS), with discrepancies resolved through discussion.

## Supplementary Information

Supplementary Information 1.

Supplementary Information 2.

## Data Availability

Data extracted from the included studies are available in a standardized Excel file, which can be found in the Supplementary Material.
