# Supplementary material for: Open-label placebos—a systematic review and meta-analysis of experimental studies with non-clinical samples
Source: Sci Rep. 2023 Mar 4;13:3640. doi: 10.1038/s41598-023-30362-z (PMC9985604; doi:10.1038/s41598-023-30362-z)
Supplement: Supplementary file 2 — Supplementary Information 2. [file 41598_2023_30362_MOESM2_ESM.docx]

**Open-label placebos: A systematic review and meta-analysis of experimental studies with non-clinical samples**

**Supplemental Material**

**Table S1**

*Studies excluded in full-text screening*

| **#** | **Title** | **Authors** | **Year** | **Reason** |
| --- | --- | --- | --- | --- |
| 1 | Open-label placebo response - Does optimism matter? A secondary-analysis of a randomized controlled trial | Locher et al. | 2019 | secondary analysis |
|  |  |  |  |  |
| 2 | The effects of a placebo and suggestion on blood pressure, heart rate, well-being and cognitive performance | Walach et al. | 2002 | no data provided upon request |
|  |  |  |  |  |
| 3 | The effects of a placebo and suggestion on blood pressure, heart rate, well-being and cognitive performance | Walach et al. | 2002 | duplicate |
|  |  |  |  |  |
| 4 | Deceptive but not open label placebos attenuate motion-induced nausea | Barnes et al. | 2019 | no data provided upon request |
|  |  |  |  |  |
| 5 | The role of patient beliefs in open-label placebo effects | Leibowitz et al. | 2019 | no suitable comparator |
|  |  |  |  |  |
| 6 | Effects of Open- and Closed-Label Nocebo and Placebo Suggestions on Itch and Itch Expectations | Meeuwis et al. | 2019 | no suitable comparator |
|  |  |  |  |  |
| 7 | Minimum clinically significant VAS differences for simultaneous (paired) interval serial pain assessments | Yamamoto et al. | 2003 | no suitable comparator |
|  |  |  |  |  |
| 8 | Effects of caffeine, caffeine-associated stimuli, and caffeine-related information on physiological and psychological arousal | Mikalsen et al. | 2001 | no suitable comparator |
|  |  |  |  |  |
| 9 | Revelation of a personal placebo response: its effects on mood, attitudes and future placebo responding | Chung et al. | 2007 | no suitable OLP condition |
|  |  |  |  |  |
| 10 | Deception: Is it a necessary component of the placebo response? An empirical investigation of the role of deception in placebo analgesia | / | / | no fulltext found |
|  |  |  |  |  |
| 11 | Effects of personal placebo response information on future placebo response | Chung | 2007 | duplicate |
|  |  |  |  |  |
| 12 | A comparison of open-label and deceptive placebo analgesia in a healthy sample | Disley et al. | 2021 | no RCT |
|  |  |  |  |  |
| 13 | Can an Open-Label Placebo Be as Effective as a Deceptive Placebo? Methodological Considerations of a Study Protocol | Druart et al. | 2020 | not finished yet (study protocol) |

**Table S1 continued**

*Studies excluded in full-text screening*

| **#** | **Title** | **Authors** | **Year** | **Reason** |
| --- | --- | --- | --- | --- |
| 14 | The Influence of Expectation on Nondeceptive Placebo and Nocebo Effects | Wei et al. | 2017 | no suitable comparator |
|  |  |  |  |  |
| 15 | Open- and Closed-Label Placebo and Nocebo Suggestions About a Sham Transdermal Patch | Meeuwis et al. | 2021 | no suitable comparator |
|  |  |  |  |  |
| 16 | Effects of open-label placebos on test performance and psychological well-being in healthy medical students: a randomized controlled trial | Kleine-Borgmann et al. | 2021 | no data provided upon request |
|  |  |  |  |  |
| 17 | Anxiety and placebos without deception: open-label placebos reduce test anxiety and improve self-management skills-a randomized controlled trial | Schaefer et al. | 2019 | duplicate |
|  |  |  |  |  |
| 18 | Advancing the viability of nondeceptive placebos to improve physical and psychological health | / | / | no fulltext found |
|  |  |  |  |  |
| 19 | No Acute Effects of Placebo or Open-Label Placebo Supplementation on Strength and Neuromuscular Fatigue | Swafford et al. | 2019 | duplicate |
|  |  |  |  |  |
| 20 | Effect of Open-placebo Intervention on Cycling Performance | Gualano | 2019 | duplicate (registration) |
|  |  |  |  |  |
| 21 | Pain Response to Open Label Placebo in Induced Acute Pain in Healthy Male Adults | Schneider et al. | 2020 | duplicate |
|  |  |  |  |  |
| 22 | Do placebo effects work when subjects know that they receive a placebo? Effects of open-label verbal suggestions on itch | Meeuwis et al. | 2017 | no fulltext found |
|  |  |  |  |  |
| 23 | Taking Open Label Placebo Further: randomized Controlled Trial of Imaginary Pills in Test Anxiety | Locher et al. | 2020 | not finished yet (registration) |
|  |  |  |  |  |
| 24 | Investigating Hope and Expectations in Open-Label Placebos | Kube | 2018 | duplicate (registration) |
|  |  |  |  |  |
| 25 | Open-label placebos and test anxiety. A randomized controlled study | Schaefer | 2018 | duplicate (registration) |
|  |  |  |  |  |
| 26 | Open-label placebos and eating behaviour. A randomized controlled study | Schaefer | 2021 | not finished yet (registration) |

**Table 1 continued**

*Studies excluded in full-text screening*

| **#** | **Title** | **Authors** | **Year** | **Reason** |
| --- | --- | --- | --- | --- |
| 27 | Influence of rituals on open-label placebo effect in healthy volunteers | Faasse | 2020 | not finished yet (registration) |
|  |  |  |  |  |
| 28 | Components of Placebo Effects in Sadness | Kube | 2018 | no fulltext found |
|  |  |  |  |  |
| 29 | The influence of open-label placebos on cognitive performance | Bingel et al. | 2019 | not finished yet (registration) |
|  |  |  |  |  |
| 30 | Impact of Open Label Placebo Administration on Cognition and Well-being | Bingel et al. | 2017 | duplicate (registration) |
|  |  |  |  |  |
| 31 | Back to Basics - Effects, Narratives and Routes of Administration of Open-label Placebo | Gaab | 2015 | duplicate (registration) |
|  |  |  |  |  |
| 32 | The non-concealed placebo: a randomized trial on smoking cessation | / | / | no fulltext found |
|  |  |  |  |  |
| 33 | Do placebo and nocebo effects work when subjects know that they receive a placebo? Effects of open-label positive and negative verbal suggestions on itch | Meeuwis et al. | 2018 | duplicate (conference abstract) |
|  |  |  |  |  |
| 34 | Effect of acupuncture and instruction on post-exercise recovery: a balanced-placebo controlled trial | Urroz et al. | 2013 | duplicate (conference abstract) |
|  |  |  |  |  |
| 35 | Open-label placebos and electromagnetic hypersensitivity. A randomized controlled trial | Schaefer | 2020 | not finished yet (registration) |

*Note.* Abbreviations: VAS = visual analogue scale, RCT = randomized controlled trial.

**Table S2**

*Detailed description of placebo specific instructions for intervention groups*

| **Study** | **Open-label placebo** | **Placebo-specific instructions** | |
| --- | --- | --- | --- |
| El Brihi et al. (2019) | “The experimenter first explained clearly that the placebo pills were inert, with no active ingredient, like sugar pills.” | “This information was followed by four discussion points outlined by Kaptchuk et al. Briefly, these were as follows: (a) The placebo effect is powerful, and placebos have been shown in numerous clinical trials to generate real physiological effects; (b) your body can automatically respond to taking placebo pills to activate mind–body healing processes; (c) positive expectations can help but are not crucial; and (d) that taking the pills as prescribed is important.” | |
|  |  |  |  |
| Glombiewski et al. (2019) | “The placebo control group was told that they would get a placebo spray, that will have no effects on mood.” | No additional instructions provided. | |
|  |  |  |  |
| Guevarra et al. (2020) | Experiment 1: “...you will be given a placebo nasal spray to reduce your negative emotional reactions. Again, this is a placebo, which means it does not contain any active ingredients, and it is completely harmless.”  Experiment 2: “From what you have read, you know that placebos are inert substances or procedures…” “I just want to remind you that I just administered a placebo nasal spray that contains no active ingredients … .” | Used in both experiments: “To maximize our persuasion attempt, our non-deceptive placebo manipulation consisted of an article reading accompanied by a verbal suggestion from the experimenter. Borrowing from Kaptchuk and colleagues’ seminal study on placebos without deception, we attempted to convey the following points about placebos: 1) placebos are powerful and work in many domains, 2) placebos can affect behavior and biology, and 3) placebos have been shown to work even when people know they are taking a placebo (i.e., without deception).” Used specifically in experiment 1: “But as you have read from the article, if you believe that the nasal spray will reduce your negative emotional reactions, then it actually will.“  Used specifically in experiment 2: “From what you have read, you know that placebos are inert substances or procedures that make people feel better mostly because they believe it will. You also know that placebos are powerful and can help reduce pain, depression, anxiety, and negative emotions. On top of that, you’ve read that placebos affect more than how you feel, they actually can change your behavior, physiology, and even brain activity. And new research has also shown that placebos can work even if you know you are taking one since the key ingredient is the positive belief that it can help and that it works.“ “I just want to remind you that I just administered a placebo nasal spray that contains no active ingredients, but if you believe it will reduce your negative emotional reactions to these images, then it will. The placebo really works because of your positive beliefs and expectations.” | |

**Table S2 continued**

*Detailed description of placebo specific instructions for intervention groups*

| **Study** | **Open-label placebo** | **Placebo-specific instructions** |
| --- | --- | --- |
| Kube et al. (2020) | “The cream you are going to receive is a placebo cream that the actual lidocaine cream is compared with.“ | “Several scientific studies have shown that placebos are very effective, even if participants knew that they were going to receive a placebo. In particular, placebo creams lead to substantial pain reduction in ∼70% of participants. Similar to Pavlov’s dogs, a placebo cream that looks like an actual analgesic cream can activate automatic bodily reactions, which in turn may lead to an effective analgesia. Thus, placebos actually affect physical processes, for example, immune parameters. Therefore, you may become less sensitive to painful stimuli after applying the cream compared to in the first trial.” |
|  |  |  |
| Locher et al. (2017) | “You are receiving a placebo cream. This means that your cream does not contain any pharmacological ingredient, hence it is an inert substance.” | “In accordance with Kaptchuk et al., the investigator stated that (1) the placebo effect is powerful, explaining that “it is well known that placebos are very effective, particularly in the area of pain, Parkinson’s disease, depression, migraine, and asthma” and described findings of placebo analgesia and open-label placebo studies. Furthermore, the investigator mentioned that (2) the “body can automatically respond to placebos like Pavlov’s dogs who salivated when they heard a bell” after explaining the classic conditioning theory. In addition, “researchers assume that this culturally anchored ritual activates automatic self-healing processes, which in turn may lead to an effective analgesia.” Finally, the investigator stated that (3) “an advantage of placebos is that a positive attitude can be helpful but is not necessary.” |
|  |  |  |
| Mathur et al. (2018) | “Participants in the placebo condition were provided with a 20 mL plastic vial labelled “Placebos” containing 40 tablets… Participants were told that the tablets did not contain active ingredients.” | “Studies have shown the placebo effect to be very powerful. This study is to determine whether the placebo effect can help heal wounds faster. A placebo is any type of treatment, like a pill, that doesn’t contain any drugs or active ingredients. The placebo effect works because of two processes: conditioning and expectations. Over the course of our lives, we have all been to the doctor and been given pills, so our body learns to associate pills with healing, which is conditioning. The other way placebos work is through our expectations. Research shows that when we expect treatments to help, they are much more likely to be effective. Since your brain and immune system are highly connected, previous learning and expectations have a big influence on your immune functioning. For this experiment, we’re testing how effective placebos are for wound healing.” |

**Table S2 continued**

*Detailed description of placebo specific instructions for intervention groups*

| **Study** | **Open-label placebo** | **Placebo specific instructions** |
| --- | --- | --- |
| Meeuwis et al. (2017) | No information provided. | “Previous research indicates that the test elicits little or no itch in most healthy people, meaning in 95% of cases. We would also like to give you some extra information. From research we know that expectations play a large role in how itch is experienced, for example through giving information about what to expect from a test such as this one. I just told you that the test that you are about to do elicits little or no itch in most healthy people. From research we know that this suggestion will really cause people to experience little itch, even when they are aware of receiving this suggestion. Thus, the suggestion alone that the test causes little or no itch will already cause you to experience little itch.” |
|  |  |  |
| Mundt et al. (2016) | “Open-label or non-deceptive placebo manipulation consisted of verbally manipulating participants’ expectations for pain reduction by instructing them that the Bivaricane cream was a placebo containing no medication.” | “However, they were also instructed that the cream is known to significantly reduce pain in some people when used in a paradigm combining classical conditioning and enhanced expectations for pain relief. The participants were thus informed that they would repeat thermal pain testing but that the experimenter would lower the intensity of the thermal stimuli on the sites where Bivaricane was applied so as to yield the perception of diminished pain following application of the Bivaricane. This procedure was described to participants as an analgesic placebo conditioning paradigm and included a brief explanation of how classical conditioning works.“ |
|  |  |  |
| Rathschlag and Klatt (2021) | “In the OPR+ group as well, the participants were told that their bottle did not contain any pharmacological substances and was, therefore, a placebo.” | “However, in addition to this, the investigator told them that the placebo effect is usually very powerful, even if people know that it is a placebo (OLP) and gave some examples from previous studies where participants benefited from a placebo. The investigator also mentioned that placebos can activate physical and mental well-being and a positive attitude can be helpful in such a process. All the participants were told: “We want to use bottles with water as a placebo which might be helpful to enhance your subjective physical and mental well-being.” |
|  |  |  |
| Rief and Glombiewski (2012) | “… participants were randomized and were informed that they were in a placebo group.” | No additional instruction provided. |

**Table S2 continued**

*Detailed description of placebo specific instructions for intervention groups*

| **Study** | **Open-label placebo** | **Placebo-specific instructions** |
| --- | --- | --- |
| Saunders et al. (2019) | “All participants were given an individual ~5-min talk in which the concept of open-placebo was explained in detail using a PowerPoint presentation.“ | “The presentation ended with three considerations for the athlete prior to taking the capsule: i) believing is important for the placebo effect; ii) however, belief in the placebo effect is not necessary as the effect may be automatic/unconscious; iii) taking the pills is important to obtain an effect.” |
|  |  |  |
| Schaefer et al. (2019) | “They were explained that placebos are inactive substances and that they contain no medications …” | “… placebo effects may still be powerful. They were told that the body may automatically respond to taking placebo pills, like Pavlov’s dogs that salivated when they heard the bell. A positive attitude may be helpful for the placebo effect, but is not necessary. Last, they were told that those participants who were in the placebo group needed to take the placebos faithfully.” |
|  |  |  |
| Schaefer et al. (2021) | “They were explained that placebos are inactive substances and that they contain no medications…” | “They were explained that placebos are inactive substances and that they contain no medications, but both deceptive as well as non-deceptive placebos may still be powerful. We further explained to them that a possible mechanism for placebo response may be classical conditioning; similar to Pavlov’s dogs that salivated when they heard the bell. In addition, we told them that a positive attitude may be helpful for the placebo effect but is not necessary. Finally, we stressed that taking the placebo pills faithfully is important.” |
|  |  |  |
| Schneider et al. (2006) | “Participants in the treatment group “True information” were told that they were to consume a “very strongly” dosed cup of black and unsweetened decaffeinated coffee.” | No additional instructions provided. |

**Table S2 continued**

*Detailed description of placebo specific instructions for intervention groups*

| **Study** | **Open-label placebo** | **Placebo-specific instructions** |
| --- | --- | --- |
| Schneider et al. (2020) | “I will now inject a placebo in your vein. As you already know from the study documentation, a placebo does not contain an active medical component.“ | “The content included (1) data regarding the strength of a placebo effect, (2) the possibility of an autonomous response of the body to a placebo, (3) a statement that, although helpful, a positive attitude toward placebo is not necessary, and (4) a television news report (original English language) with German translation (subtitles) pertaining to open label placebo. We know from recent research that placebos can have a strong positive effect on pain. I am confident that this placebo will substantially reduce your pain as well.” |
|  |  |  |
| Swafford et al. (2020) | “For the OLP trial, participants were told that they would be consuming capsules that had no active ingredients ….” | “… clinical studies have shown that OLP treatments enhance function and minimize pain”. |
|  |  |  |
| Urroz et a. (2016) | “Participants randomized to receive A-Lo or P-Lo were informed that they would be receiving placebo acupuncture.” | No additional instructions provided. |
|  |  |  |
| Walach et al. (2001) | “Subjects received a caffeine placebo and were told so.” | “After having provided the "coffee" to the subjects, given the consumption instructions, the experimenter left the subjects to themselves.” |

*Note.* The same instructions were used in both experiments described in Rathschlag & Klatt (2021).

**Table S3**

*Effect sizes and statistical inferences for subgroups with different amounts of suggestiveness*

| Meta analysis | Moderator | Subgroup | k | SMD | 95% CI | p | Q |
| --- | --- | --- | --- | --- | --- | --- | --- |
| Self-report | Suggestiveness of instructions | 0 statement | 3 | 0.33 | [0.03; 0.63] | .03 | 1.52 |
|  |  | 1 statement | 4 | 0.55 | [0.24; 0.86] | < .001 | 3.87 |
|  |  | 2 statements | 2 | 0.38 | [-0.22; 0.97] | .21 | 2.23 |
|  |  | 3 statements | 4 | 0.41 | [0.13; 0.70] | < .01 | 4 |
|  |  | Difference |  |  |  | .80 | 1.02 |
| Objective | Suggestiveness of instructions | 0 statement | 4 | -0.25 | [-0.54; 0.04] | .09 | 0.90 |
|  |  | 1 statement | 1 | 0.38 | [0.09; 0.66] | < .01 | - |
|  |  | 2 statements | 2 | -0.07 | [-0.56; 0.41] | .76 | 1.73 |
|  |  | 3 statements | 1 | 0.09 | [-0.36; 0.54] | .69 | - |
|  |  | Difference |  |  |  | .02 | 9.49 |

Abbreviations. k = number of studies

**Table S4**

*Effect sizes and statistical inferences for subgroups of different control conditions*

| Meta analysis | Moderator | Subgroup | k | SMD | 95% CI | p | Q |
| --- | --- | --- | --- | --- | --- | --- | --- |
| Self-report | Type of control | CP | 2 | 0.85 | [0.46; 1.24] | < .001 | 0.59 |
|  |  | NT | 11 | 0.36 | [0.21; 0.52] | < .001 | 7.08 |
|  |  | Difference |  |  |  | .02 | 5.26 |
| Objective | Type of control | CP | 1 | 0.38 | [0.09; 0.66] | < .01 | - |
|  |  | NT | 7 | -0.13 | [-0.33; 0.07] | .021 | 4.26 |
|  |  | Difference |  |  |  | < .01 | 8.14 |

Abbreviations. k = number of studies, CP = covert placebo, NT = no treatment

**Table S5**

*Effect sizes and statistical inferences for subgroups of different types of studies*

| Meta analysis | Moderator | Subgroup | k | SMD | 95% CI | p | Q |
| --- | --- | --- | --- | --- | --- | --- | --- |
| Self-report | Type of study | Clinically orientated studies | 4 | 0.53 | [0.25; 0.81] | < .001 | 1.66 |
|  |  | Lab studies | 9 | 0.39 | [0.19; 0.58] | < .001 | 10.58 |
|  |  | Difference |  |  |  | .41 | 0.68 |

Abbreviations. k = number of studies

**Table S6**

*Overall Quality of Evidence Profile using the Grading of Recommendations Assessment, Development and Evaluation System*

| No of studies | Quality assessment | | | | | | | Number of participants | | Effect | Quality |
| --- | --- | --- | --- | --- | --- | --- | --- | --- | --- | --- | --- |
|  | Study design | Risk of bias | Inconsistency | Indirectness | Imprecision | Publication bias | Upgrading factors | OLP | NT/CP |  |  |
| **Self-reported pain** | | | | | | | | | | | |
| 3 | RCT | Serious (-1) | Not serious | Serious (-1)  unrepresentative  population  (mostly students) | Very serious (-2)  Small sample, CI includes no effect and benefit | Undetected | None | 87 | 90 | SMD = 0.29 [-0.06; 0.64] | ⊕⊝⊝⊝  Very low |
| **Objective pain** | | | | | | | | | | | |
| 3 | RCT | Serious (-1) | Not serious | Not serious | Serious (-1)  Small sample, inconsistent CI | Undetected | None | 103 | 85 | SMD = 0.00 [-0.30; 0.29] | ⊕⊕⊝⊝  low |
| **Self-reported positive well-being** | | | | | | | | | | | |
| 6 | RCT | Serious (-1) | Not serious | Not serious | Serious (-1)  Small sample, upper CI crosses SMD of 0.5 | Undetected | None | 184 | 140 | SMD = 0.53 [0.30; 0.76] | ⊕⊕⊝⊝  low |

**Table S6 continued**

*Overall Quality of Evidence Profile using the Grading of Recommendations Assessment, Development and Evaluation System*

| No of studies | Quality assessment | | | | | | | Number of participants | | Effect | Quality |
| --- | --- | --- | --- | --- | --- | --- | --- | --- | --- | --- | --- |
|  | Study design | Risk of bias | Inconsistency | Indirectness | Imprecision | Publication bias | Upgrading factors | OLP | NT/CP |  |  |
| **Self-reported distress** | | | | | | | | | | | |
| 7 | RCT | Serious (-1) | Not serious | Not serious | Serious (-1)  Small sample, upper CI crosses SMD of 0.5 | Undetected | None | 213 | 182 | SMD = 0.46 [0.24; 0.68] | ⊕⊕⊝⊝  low |
| **Physiological outcomes (Urroz et al.)** | | | | | | | | | | | |
| 1 | RCT | Serious (-1) | Not serious (N.A), single study | Serious (-1)  unrepresentative  population  (young adults, sportive) | Very serious (-2)  Very small sample, wide CI | Undetected (N.A), single study | None | 12 | 12 | SMD = 0.10 [-0.71; 0.90] | ⊕⊝⊝⊝  Very low |
| **Physiological outcomes (Wallach et al.)** | | | | | | | | | | | |
| 1 | RCT | Not serious | Not serious (N.A), single study | Serious (-1)  unrepresentative  population  (mostly students) | Very serious (-2)  Very small sample, wide CI | Undetected (N.A), single study | None | 41 | 37 | SMD = -0.32  [-0.77; 0.13] | ⊕⊝⊝⊝  Very low |
| **Physiological outcomes (Schneider et al.)** | | | | | | | | | | | |
| 1 | RCT | Serious (-1) | Not serious (N.A), single study | Not serious | Very serious (-2)  Very small sample, wide CI | Undetected (N.A), single study | None | 15 | 15 | SMD = -0.21 [-0.94; 0.53] | ⊕⊝⊝⊝  Very low |

*Note.* Quality of evidence rating for different clusters of outcomes. The overall quality of evidence is rated as high, moderate, low or very low. RCTs have high quality of evidence to begin with. This rating can be downgraded as a function of five factors, namely risk of bias, indirectness, inconsistency, imprecision, and publication bias. The grading can also be upgraded as a function of three additional factors based on the magnitude of the effect, dose response, and the effect of any plausible confounders. Abbreviations: OLP = open-label placebo, NT = no treatment, CP = covert placebo, SMD = standardized mean difference, N.A = not applicable.

**Table S7**

#### Search Strategy in Medline via PubMed

| Step | Searches | Results |
| --- | --- | --- |
| #1 | placebos[Title/Abstract] | 3,447 |
| #2 | placebo*[Title] | 38,924 |
| #3 | #1 OR #2 | 41,023 |
| #4 | told[Title/Abstract] OR nondecept*[Title/Abstract] OR "non decept*"[Title/Abstract] OR nonconceal*[Title/Abstract] OR "non conceal*" [Title/Abstract] OR nonblind*[Title/Abstract] OR "non blind*"[Title/Abstract] OR unblind*[Title/Abstract] OR "without deception"[Title/Abstract] OR "without conceal*"[Title/Abstract] OR "without blind*"[Title/Abstract] | 16,314 |
| #5 | #3 AND #4 | 317 |
| #6 | "open placebo*"[Title/Abstract] OR "open label placebo*"[Title/Abstract] | 184 |
| #7 | #5 OR #6 | 487 |

**Table S8**

#### Search Strategy in The Cochrane Central Register of Controlled Trials (CENTRAL)

| Step | Searches | Results |
| --- | --- | --- |
| #1 | (placebos):ti,ab,kw | 26,407 |
| #2 | (placebo*):ti | 72,396 |
| #3 | # 1 OR #2 | 92,355 |
| #4 | (told OR nondecept* OR "non decept*" OR nonconceal* OR "non conceal*" OR nonblind* OR "non blind*" OR unblind OR "without deception" OR "without conceal*" OR "without blind*"):ti,ab,kw | 8,880 |
| #5 | #3 AND #4 | 620 |
| #6 | (open OR “open label”) NEAR/1 placebo*:ti,ab,kw | 420 |
| #7 | #5 OR #6 | 1,011 |

**Table S9**

#### Search Strategy in PsycINFO via EBSCO

| Step | Searches | Results |
| --- | --- | --- |
| #1 | TI Placebos OR AB Placebos | 41,183 |
| #2 | TI Placebo* | 8,060 |
| #3 | # 1 OR #2 | 41,184 |
| #4 | TI (told OR nondecept* OR "non decept*" OR nonconceal* OR "non conceal*" OR nonblind* OR "non blind*" OR unblind* OR "without deception" OR "without conceal*" OR "without blind*") OR AB (told OR nondecept* OR "non decept*" OR nonconceal* OR "non conceal*" OR nonblind* OR "non blind*" OR unblind* OR "without deception" OR "without conceal*" OR "without blind*") | 14,157 |
| #5 | #3 AND #4 | 409 |
| #6 | TI ((open OR “open label”) N1 Placebo*) OR AB ((open OR “open label”) N1 Placebo*) | 91 |
| #7 | #5 OR #6 | 494 |

**Table S10**

#### Search Strategy in Web of Science Core Collection

| Step | Searches | Results |
| --- | --- | --- |
| #1 | TI=Placebos OR AB=Placebos | 242,618 |
| #2 | TI=Placebo* | 56,702 |
| #3 | # 1 OR #2 | 242,680 |
| #4 | TI=(told OR nondecept* OR "non decept*" OR nonconceal* OR "non conceal*" OR nonblind* OR "non blind*" OR unblind* OR "without deception" OR "without conceal*" OR "without blind*") OR AB=told OR nondecept* OR "non decept*" OR nonconceal* OR "non conceal*" OR nonblind* OR "non blind*" OR unblind* OR "without deception" OR "without conceal*" OR "without blind*") | 86,825 |
| #5 | #3 AND #4 | 1,366 |
| #6 | TI=("open placebo*" OR "open label placebo*") OR AB=("open placebo*" OR "open label placebo*") | 195 |
| #7 | #5 OR #6 | 1,543 |

**Table S11**

#### Search Strategy in Psyndex via EBSCO

| Step | Searches | Results |
| --- | --- | --- |
| #1 | TI Placebos OR AB Placebos | 1,915 |
| #2 | TI Placebo* | 470 |
| #3 | # 1 OR #2 | 1,917 |
| #4 | TI (told OR nondecept* OR "non decept*" OR nonconceal* OR "non conceal*" OR nonblind* OR "non blind*" OR unblind* OR "without deception" OR "without conceal*" OR "without blind*") OR AB (told OR nondecept* OR "non decept*" OR nonconceal* OR "non conceal*" OR nonblind* OR "non blind*" OR unblind* OR "without deception" OR "without conceal*" OR "without blind*") | 387 |
| #5 | #3 AND #4 | 25 |
| #6 | TI ((open OR “open label”) N1 Placebo*) OR AB ((open OR “open label”) N1 Placebo*) | 13 |
| #7 | #5 OR #6 | 35 |

**Figure S1**

*Risk of bias assessment of included studies according to RoB 2*

**R D Mi Me S O**

**
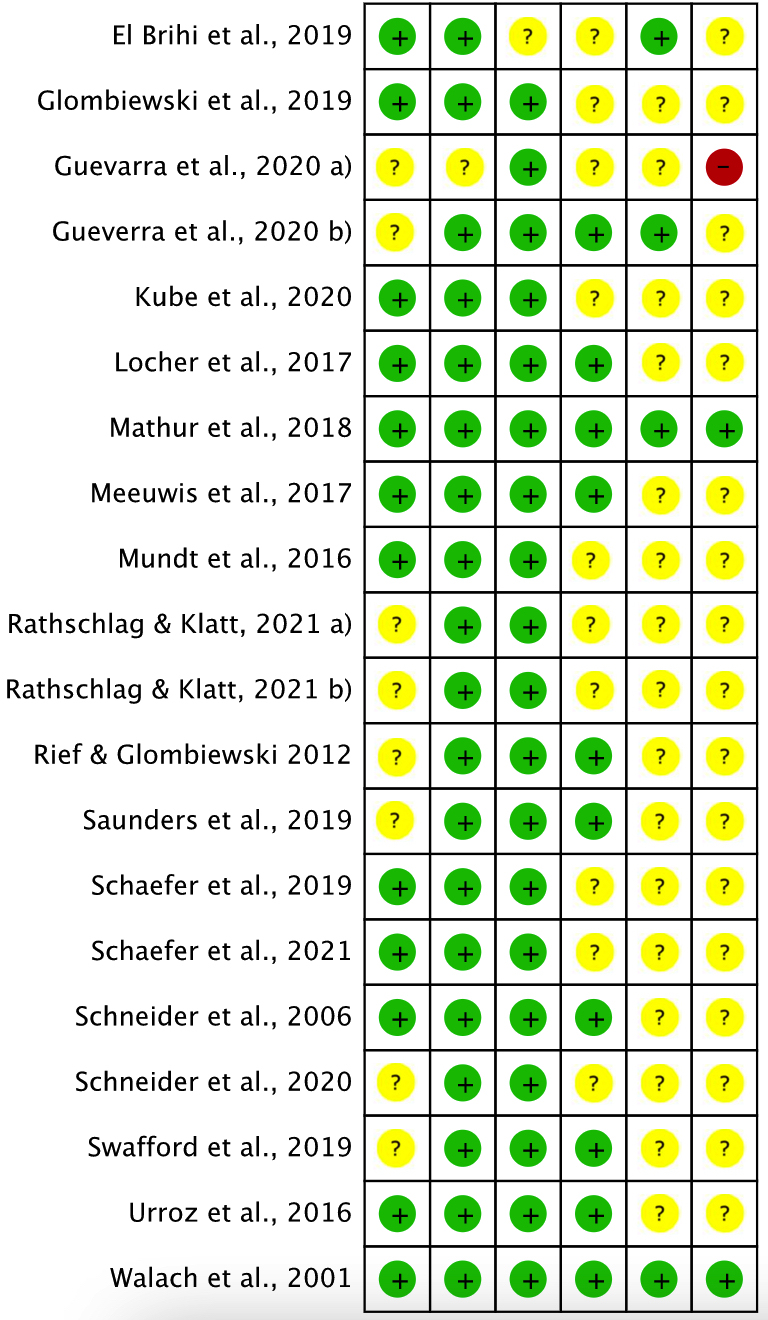
**

*Note.* The colors represent the risk of bias, with green indicating low risk, yellow indicating some concerns, and red indicating high risk of bias. R = risk of bias arising from the randomization process, D = risk of bias due to deviations from the intended interventions, Mi = risk of bias due to missing outcome data, Me = risk of bias in measurement of the outcome, S = risk of bias in selection of the reported result, O = overall risk of bias.
